# Effect of institutional mechanisms on micropension saving among informal economy workers in the Greater Accra Region of Ghana

**DOI:** 10.1016/j.heliyon.2021.e08004

**Published:** 2021-09-16

**Authors:** Dominic Buer Boyetey, Owusu Boampong, Francis Enu-Kwesi

**Affiliations:** a Department of Sustainable Development, University of Environment and Sustainable Development, Somanya, Ghana; b Department of Integrated Development Studies, University of Cape Coast, Ghana

**Keywords:** Economy, Financial, Informal, Institutions, Micropension, Security, Social, Workers

## Abstract

The study investigated the effect of institutional mechanisms of micropension saving (MPS) schemes in extending coverage to informal economy workers. We used mixed methods as the research approach, and collected both quantitative and qualitative data for analysis. Using principal component analysis, multiple regression analysis and interpretative approaches that yielded themes, we concluded that more access provision, incentives and security result in increased informal economy workers’ participation in MPS. However, general form of financial information to informal economy workers was found to demotivate enrolment onto the scheme. Consequently, we recommended that corporate pension trustees should create institutional structures like pension education campaigns on national television and radio to promote the culture of pension saving.

## Teaser

Informal economy; Financial institutions; Micropension; Security social; Workers.

## 1 Introduction

Reports across the globe suggest a surge in informal economy workforce, with statistics from International Labour Organisation (ILO) (2018) indicating that informal employment excluding agriculture in Africa is 80%, while those for Latin America and Asia are 55% and 85% respectively. These large segments of the labour force make the delivery of Micropension Saving (MPS) appropriate in order to forestall any risk of workers falling into extreme poverty, which is consistent with Goal One of the United Nation's Sustainable Development Goals (United Nations [UN], 2017). MPS, as explained by Shankar and Asher (2009), is a voluntary contribution scheme where savings are invested through financial and capital markets by a professional fund manager over an extended period and paid, either in a lump sum, a phased withdrawal, annuity or some combination of these options.

The literature on micropension saving schemes is largely informed by the Life Cycle Hypothesis (LCH) and the Institutional Theory of Savings (ITS). These theories propose strategies for the avoidance of poverty post-retirement and in old-age (Agravat and Kaplelach, 2017). Central to the LCH, as Chipote and Tsegaye (2014) observed, is the fact that rational individuals maximise satisfaction of their future consumption by saving to finance their retirement consumption and dissaving during retirement. Njunge (2013) highlights the relevance of the LCH to MPS and maintains that through micropension savings, individuals could transfer purchasing power from one phase of their life to another. This can affect how they live after retirement. With respect to ITS, Schreiner and Sherraden (2007) postulate that an individual's financial or real asset accumulation which leads to income security in retirement is dependent upon the access, incentives, information, facilitations, expectations, restrictions and securities afforded by institutions.

MPS schemes built on these institutional factors according to Barr and Diamond (2009), offer safety nets to informal workers to save and avoid risk of old-age poverty. These remove all forms of barriers that constrain income-risk informal workers from participating in the social security schemes of formal economy workers (Bucheli et al., 2010; Bosch and Manacorda, 2012). Micropension saving scheme, therefore, compensates for the gaps in contributory social security coverage and reduces the vulnerability of low-income persons and informal economy workers to help them to better manage risks and combat economic insecurities (Mukherjee, 2014).

Different countries have varying forms of micropension systems for the benefit of informal economy workers. In China, Hu and Stewart (2009) observed that the scheme is characterised by minimal compulsion as well as voluntary private pension that attracts many small-scale workers due to simplification and flexibility features of the scheme. In Bangladesh and India, Goyal (2010) reports that the Partner Agent Model (PAM) and the Grameen model are the most common voluntary pensions in operation. In the case of Chile, the Organization for Economic Co-operation and Development (OECD) (2013) reports a blend of voluntary schemes with government subsidies and co-sponsored schemes allowing for flexible contributions and withdrawal terms as well as tax-free incentive on contributions and investment income.

Ghana, with a large informal economy workforce and a fast-growing aging population (Ghana Statistical Service [GSS], 2015; World Health Organization [WHO], 2014), has introduced MPS as a strategy to secure the financial stability of informal workers when they are old and no longer able to participate in the labour market. Ghana's MPS scheme is a voluntary scheme (also known as the tier-three pension scheme) established by law, Act 766 (National Pension Act, 2008). The Act provides that workers in the informal economy, just like their counterparts in the formal economy, will also receive monthly pensions or a lump sum after retirement. This is particularly important for the informal economy workers in the Greater Accra region where 84.1% of the people are engaged in informal employment activities (Ghana Statistical Service, 2015).

According to Kumah et al. (2017), the introduction of the scheme in Ghana has provided pension coverage to the informal population who were previously difficult to cover through the formal economy pension approaches, and thus at risk of old-age poverty. Collins-Sowah et al. (2013) are of the view that people in the informal economy cherish voluntary saving products like micropensions due to the economic security it promises. In this regard, the introduction of the tier-three pension scheme affords workers in the informal economy an opportunity to save towards retirement (Afenyadu, 2014).

Some studies have touted the benefits of MPS among informal economy workers in Ghana. Asante (2016) gave an indication that basic pension benefits for MPS positively affect households with elderly members, and by extension, provides a reduction in aggregate poverty (Ping, 2013). Evidence from Northern Ghana, as reported by Adzawla et al. (2015), indicates that informal pension scheme provides economic security to people who wish to satisfy their basic needs both presently and in the future. Adzawla et al. concluded that the tier-three MPS is an essential mechanism for a sustained poverty eradication and development. Darko (2016) also observed that the third tier MPS concept is better in addressing inadequacies and discriminations found in the state-sponsored pension schemes when it comes to providing old-age income security to low income earners.

However, studies (Adzawla et al., 2015; Kumah et al., 2017) on the coverage level of the pension system among informal workers in Ghana, especially with the coming into being of the new pension law, Act 766 (2008), suggest that the level of pension coverage among the informal economy workforce is minimal. Thus, close to 10 years after the establishment of the tier-three MPS scheme, the adoption rate has only been about 2% which implies that informal economy workers are completely at a higher risk of adverse conditions and unavoidable income insecurity at old age (Wireko-Brobby, 2018; Ministry of Employment and Labour Relations [MELR], 2017).

Previous empirical studies (Afenyadu, 2014; Adzawla et al., 2015; Asante, 2016; Darko, 2016) also attribute the low coverage of MPS schemes to inaccessibility of the schemes, inadequate pension information and lack of incentives, making them the main barriers limiting informal economy workers’ enrolments in MPS schemes in Kumasi, Accra, Koforidua and Tamale Metropolis. These barriers bring into question the hypothesis of the ITS that access, incentives, information, facilitations, expectations, restrictions and securities offered by institutions lead to savings accumulation, and raise the opportunity to interrogate these institutional mechanisms involved in extending MPS coverage to informal economy workers in the Greater Accra Region of Ghana.

Consequently, we employed a mixed method research design where both quantitative and qualitative research assumptions were applied concurrently to investigate how the institutional mechanisms of MPS extend coverage to informal economy workers. This strategy was deployed because it allowed the use of diverse types of data that give best answers to the research problem (Creswell, 2009). The subsequent sections of this paper contain a review of related literature resulting in a conceptual framework, followed by the methodology and results. The last section of the paper comprises the conclusion and related policy implications.

## 2 Literature review

The literature reviewed entails discussions of the institutional theory of savings and its linkage with the life-cycle hypothesis, key concepts, empirical studies and a conceptual framework of the study. Beverly et al. (2008) explained the ITS in stimulating savings accumulation by emphasising the role of institutions in establishing formal and informal relationships with low-income workers. As much as the ITS provides an explanation of how individuals and institutions interact for pension savings accumulation, it fails to recognize the income earned and the amount to save for a secured retirement life. The LCH therefore, provides that an individual's decision to attain maximum satisfaction (income security) during retirement is premised on continuous income and a constant consumption during working life; the difference between income and consumption generates savings which accumulates as wealth to finance retirement consumption (Castel, 2006).

The paper focuses on two main conceptual issues; micropension saving and institutional mechanisms offered in MPS schemes. The terms pension scheme, retirement income maintenance and social insurance are all related words that refer to income earned by a person during the time he or she is not working, according to Wang et al. (2014). Accordingly, Mitchell and Fields (1996) and Rofman et al. (2012) described pension scheme as a contract for annuity payment to a worker who retires from work after reaching a prescribed age. The ultimate objectives of pension schemes are basically to smoothen consumption of the elderly, especially to protect them against the risk that consumption will drop when their income ceases as well as also to protect them against the risk of old-age poverty, as argued by Mackellar (2009) and Blake et al. (2010).

However, the risk associated with informal jobs, which are viewed as a survival strategy for many informal economy workers, limit their eligibility to many pension schemes (Osei-Boateng and Ampratwum, 2011). Micropension saving schemes, explained by Mukherjee (2014) and Sahu (2014) as any financial plan to keep up with old age income or consumption are, therefore, proposed as a necessary financial vehicle that secures the financial stability of informal economy workers during old-age. In Arunachalam's (2007) view, micropension saving scheme, compared to other pension schemes, has the primary objectives of reducing poverty, eliminating the risk of the fallen living standards as well as protecting the aged from economic and social crisis. Mackellar (2009) observes that the scheme contributes to the investors' economic security as their productivity and income-generating capacity decreases, while Njuguna (2012) expresses the view that the scheme fosters participants' financial independence through saving and insurance.

Moreover, special features of the scheme as engineered by financial institutions make the scheme attractive to many informal economy workers. According to Beverly and Sherraden (1999), institutional mechanisms embedded in the schemes aim at satisfying clients through the provision of access, incentives, information, facilitation, expectation, restriction and security. Schreiner and Sherraden (2007), described access as the removal of any barrier that prevents informal economy workers from partaking in a pension scheme. Deriving from this, Sinha (2009) and Uthira and Manohar (2009) explain that financial institutions increase access by making it easier to open an account, reducing fees associated with maintaining an account, awareness creation, proximity to service providers and flexible arrangement for contribution and payment. Thornton et al. (2010) also believe that these are particularly important in motivating low-income people to join MPS schemes. Sinha (2009), for instance, advocates for tax and monetary incentives such as high rate of returns to attract and encourage informal economy workers to contribute to micropension saving.

According to Turner and Manturuk (2012), incentives can be seen as financial motivators that encourage enrolment onto savings schemes, while information refers to the financial information, advice and education provided to consumers and investors regarding financial products (OECD, 2005). Beverly et al. (2003) explain that facilitation is any form of assistance and Matul et al. (2011) suggested that simplification of claim procedures, faster claim settlement, product tangibility and prompt feedback mechanisms all facilitate clients’ decision to join MPS schemes. Lambe et al. (2002) argued that expectations are embodied in institutional features such as meeting savings targets in order to enjoy some monetary incentives or mortgage arrangements, while restrictions, according to Ssewamala and Sherraden (2004) and Sherraden et al. (2005), refer to prohibitions or rules that restrict access to the use of pension savings or any other financial asset. Beverly et al. (2008) defined security as freedom from unreasonable risk in saving and asset holding. Mohd et al. (2010) and Durner and Shetret (2015) cited competence and integrity of the political system, integrity of the financial markets, management of the macro economy, trust and safety of financial institutions as key financial security risk elements capable of explaining outcomes in MPS.

Some researchers (Clancy et al., 2006; Hu and Stewart, 2009; Kwena and Turner, 2013; Schreiner and Sherraden, 2007) have paid attention to institutional mechanisms in extending MPS coverage to informal economy workers, but the results have been mixed. Studies by Han and Sherraden (2007) and Curley et al. (2009) generally concurred that the provision of institutional mechanisms has significant explanatory powers that affect the value of pension savings of low-income workers. Particularly, Curley et al. noted the statistical significance of information and expectation in shaping savings of low income workers, but access, information and facilitation were seen as insignificant. This assertion was contrary to Han and Sherranden's (2007) findings that access, facilitation and incentives significantly explained variations in saving outcomes. What was missing in these studies were the effect of institutional variables such as restriction and security.

Other researchers (Afenyadu, 2014; Adzawla et al., 2015; Onyango, 2014) have attributed for the limited micropension uptake among informal economy workers to institutional inactions. According to Afenyadu, inadequate awareness is one of the reasons for low participation, while Onyango cited mistrust of scheme providers, corruption and embezzlement of funds and schemes’ insolvency as reasons for non-participation in pension saving schemes. However, Adzawla et al. (2015) found that majority of the respondents were aware of the voluntary pension schemes, but constraints such as limited understanding, and inaccessibility were some of the major reasons why they do not contribute to the scheme.

Kumah et al.’s (2017) finding of low coverage of informal workers in pension saving schemes concords with those of Afenyadu (2014), Adzawla et al. (2015) and Ares et al. (2015). Kumah et al. attributed the reason for the low patronage of pension saving schemes to inadequate awareness of MPS schemes, similar to Afenyadu (2014). In addition, they mention unavailability of pension institutions amongst reasons culminating in non-viability and unsuitability of the scheme. Despite the new interest in the study of MPS among informal workers, existing studies in Ghana like Afenyadu's (2014), Adzawla et al.’s (2015) and Kumah et al.’s (2017) did not pay attention to the effect of institutional mechanisms of MPS in shaping retirement income of informal economy workers.

**Figure 1:**
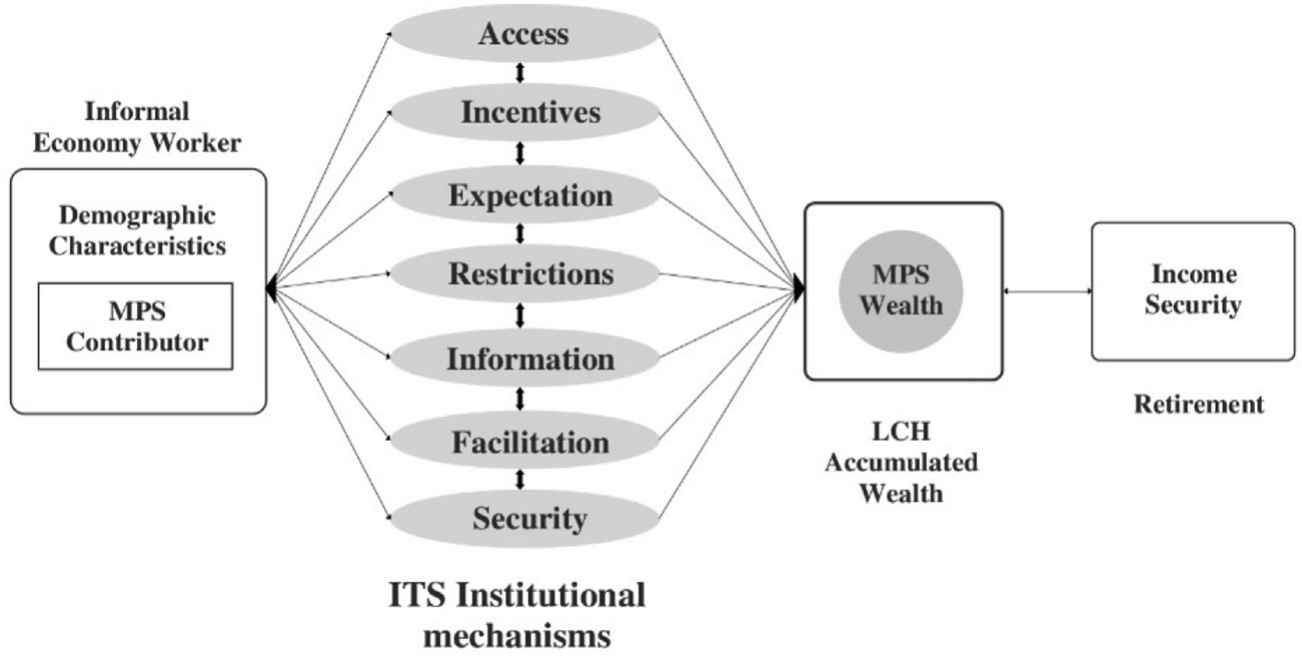
Conceptual framework of institutional mechanisms in MPS schemes. Source: Authors Construct based on reviewed literature (2019).

In our conceptual framework (Figure 1), we present the effects of institutional mechanisms in shaping accumulated pension savings and wealth for subsequent old-age income security for informal economy workers. The direction of effect can be inferred from the figure that a contributor of MPS builds-up accumulated wealth, which then becomes a buffer for old age for income security.

## 3 Methodology

### 3.1 Study setting

This study is limited to the Greater Accra Region of Ghana with a projected population of about 4,613,637 people in 2016 (GSS, 2016). The region is divided into 16 administrative districts. The region has 84.1%, of the workforce engaged in informal employment activities according to Ghana Statistical Service (2015). Most of these informal workers, according to Adzawla et al. (2015) are not covered by any pension schemes, which expose them to the risk of old-age income insecurity and poverty. In addition, major players in the provision of pension services are located in the region. Several of the institutions such as Micro Finance Institutions (MFI), investment banks and insurance companies have taken the opportunity in extending their services to the large informal market which, otherwise, are left out of the formal pension systems, and thus offering them tailored schemes.

### 3.2 Study design

We used a concurrent mixed method design, which allowed the use of both quantitative and qualitative data simultaneously (Creswell, 2009) in order to provide a comprehensive analysis of how institutional mechanisms shape pension savings accumulation. Specifically, we adopted a cross-sectional study to investigate institutional mechanisms of MPS schemes in extending coverage to informal economy workers. The cross-sectional was most appropriate because it allowed us to employ probability sampling procedures to select respondents, and interviewing as a medium to investigate the issues with the intention of generalizing at a point in time (Bryman, 2008; Creswell, 2012). Furthermore, the design created the opportunity for us to assess and subsequently test hypotheses using both quantitative and qualitative measures in data collection and analysis with respect to the institutional arrangements that shape pension savings (Afenyadu, 2014; Asante, 2016; Kumah et al., 2017).

### 3.3 Sampling

The population for the study included all informal economy workers who are contributors (IWC) of MPS with any corporate trustee licensed by the National Pension Regulatory Authority (NPRA) and Pension Scheme Administrators (PSA). Records from the NPRA showed that the total contributors to informal sector pension schemes in Ghana were 152,000 in 2018 (Wireko-Brobby, 2018). This number was aggregated from 34 licensed corporate trustees. The corporate trustees provided contact lists of informal investors in the Greater Accra Region, which formed the sampling frame for the population of IWC. The choice of these survey participants were in accordance with the assumptions of the concurrent mixed method design. The mixed participants allowed broader view of the survey in order to generalize results to the population as Creswell (2009) proposes.

A sample size of approximately 390 was estimated using Yamane's (1967) method of estimating a representative sample. The sampling procedure involved a multi-stage sampling approach. In the first stage, 30 corporate trustees were selected from the nationwide total of 34 trustees by a simple random sampling method. From each of the selected trustees, a uniform random sample allocation of IWC residents in Greater Accra Region was determined at the second stage. This became the sampling frame from which the ultimate sampling units were obtained. Thirteen participants were randomly selected from each of the 30 corporate trustees to constitute the sample size of 390. For the qualitative aspect of the study, we purposively sampled ten pension scheme administrators as key informants.

### 3.4 Data collection instruments

We used interview schedules and interview guide to collect quantitative and qualitative data respectively. The interview schedule featured closed and open-ended items that covered key issues such as amount saved for pension and perceptions about access, incentives, information, expectation, facilitation, restriction and security afforded by the scheme providers. Perception related data on respondents’ experience with the tier-three MPS scheme on key issues such as accessibility to the scheme, information provided by the scheme, incentives offered by the scheme and other institutional arrangements that may motivate/demotivate enrolment to the scheme were measured using an interval scale. The interview guide was thematized to cover similar issues as the interview schedule, and to enable triangulation of the findings of the study.

### 3.5 Pre-testing and fieldwork

We pre-tested the interview schedule by administering copies to 120 IWC in Kasoa, a peri-urban town in the Awutu Senya East Municipality of the Central Region of Ghana in October 2019. Cronbach's coefficient alpha was employed to test for internal consistency and reliability of the measures used for the study (Fariborz and Jaleh, 2013; Nunnally, 1978). The test result showed a Cronbach's alpha of 0.823 for 33 items for the interview schedule covering institutional mechanisms. Based on the reliability test results we went ahead and used the interview schedule for final data collection. With the aid of tape recorders, we also used the interview guide to collect data from the key informants, and each such interview lasted for approximately 15 min. Prior to the data collection, ethical issues such as informed consent, anonymity, confidentiality and non-disclosure were complied with. The University of Cape Coast Institutional Review Board issued an ethical clearance for this research with a reference ID of UCCIRB/CHLS/2019/19. The entire data collection occurred in November 2019.

### 3.6 Procedures and data analysis

With the help of the SPSS Version 21 software, the data were analysed by using principal component analysis (PCA) and multiple regression analysis. We initially used PCA to extract various components that define institutional mechanisms. These extracted institutional factors were used in a multiple regression model as predictors with the log of amount saved as the response variable to measure the variations in MPS enrolment. The PCA was preferred for the reason that it summarises empirical data set and reveals hidden or latent structures in the data (Costello and Orsborne, 2005) while multiple regression was applied with the focus of identifying change in variance accounted for in the dependent variable by specific independent latent variables (Cohen and Cohen, 1983). Concurrently, we used descriptive or interpretative approach to analyse the qualitative data and derived themes thereafter.

## 4 Results and discussion

The discussions are underpinned by the institutional theory of savings. We used the propositions in the ITS as the framework for investigating the institutional mechanisms of MPS schemes. We presented the descriptions of the background characteristics of respondents and followed that with the results pertaining to the institutional mechanisms. The substantive results are based on estimates from the PCA and the multiple regression analysis. The response rate for the survey was 82.31 percent. Sixty-nine of the administered instruments were found to be incomplete and were therefore discarded. The response rate was above the minimum threshold of 75 percent as required for a statistical representation of a sample frame (Saunders at al., 2011).

### 4.1 Background characteristics

As can be observed from Table 1, the study establishes that females (65%) constituted majority of the informal economy workers who contribute to MPS. A little more than half of the respondents (52%) were found to be married, while the indication is that more of the contributors of MPS seem to have at least up-to primary education (cumulatively about 90%). The results also show that most IWCs are in their productive age in life, with a modal age class of 30–39 years (42.7%). Overall, the IWC have a high dependency ratio. These demographic features were largely mentioned in previous studies (Adzawla et al., 2015; Curley et al., 2009; Han and Sherraden, 2007) to have had significant influence in shaping retirement savings of low-income earners.

**Table 1:** Demographic characteristics of IWC of MPS.

Variables	Category	Number	%
Sex	Male	112	35
	Female	209	65
Marital Status	Married	169	52
	Single	83	26
	Separated	23	7
	Divorced	9	3
	Widowed	15	5
	Living together	19	6
	Others	3	1
Education	No Education	31	10
	Up-to-Primary	40	13
	Up-to-MSLC/JHS	104	32
	Up-to-SH/SS/Tch/Vc.	133	41
	Up-to-Tertiary	13	4
Age (years)	20–29	45	14
	30–39	137	43
	40–49	90	28
	50–59	38	12
	60 & above	11	3
Dependents	No Dependent	19	6
	1–2	87	27
	3–4	135	42
	5–6	55	17
	7 & Above.	25	8
Variable N	Mean Std. Dev.	Min.	Max.
Amt saved (¢) 321	66.9315 117.6149	1.00	900.00

Source: Field survey (2019).

The pension savings’ results revealed that many of the respondents do not save regularly (45.2%). However, some of them are able to put aside a median of GH¢5.00 at a time as pension savings (mean = 66.9315; std. deviation = 117.6149; skewness = 4.019). The large variance of savings, according to OECD (2013), is as a result of fluctuating income earned in the informal economy and also due to seasonal nature of employment in the sector.

### 4.2 MPS institutional arrangements

Thirty-three items identified to motivate pension savings by informal economy workers contributing to MPS were assessed. The 33 questionnaire items were key issues raised in the reviewed literature. They comprised research findings, benefits and challenges about access, incentives, information, expectation, facilitation, restriction and security afforded by the scheme providers. The study therefore assessed IWC's perceptions about these 33 items identified consistent with procedures used in PCA (Pallant, 2005).

Using PCA, eleven components were extracted out of the 33 items (KMO = 0.784; $\chi^{2}=3658.863$, d.f=231, p-value=0.000). Cumulatively, these components accounted for approximately 80 percent of total variability in the responses. The components had eigenvalues greater than one, as required for PCA by Pallant (2005). Table 2 in Appendix A displays the factor loadings for the PCA and confirms the number of components extracted. The items in the first component tell the extent to which travel time, transaction charges, proximity to institutions, ease of opening account, eligibility criteria and awareness creation inform the decision of informal workers to enrol on MPS schemes. In the second component, it can be observed that returns on investment, complementary services, acceptable interest rate, mortgage facilities and tax exemptions are positively linearly related. Items loading high on the third and fourth components describe institutional processes and facilitations respectively.

The fifth component comprised four items, namely financial information, financial calculation, pension and contribution education. Under the sixth component is a hidden variable called specific financial information. The two items under the specific financial information include information on pension benefit and calculation of investment return. Components seven and eight have a common theme, dubbed, “expectation of reward”. Going forward in the discussion, the underlying latent variables in components nine, ten and eleven are financial institution security, economy wide security and safe institutional rules respectively. In the financial institution security, confidence in institutions correlates positively with integrity in the financial market/institution. The economy-wide security is made up of positive linear combination of sound management of the economy and integrity in political systems. Finally, safe institutional rules are defined by a combination of acceptable saving rules and a safer financial institution.

Out of the eleven latent variables, five namely, access provision, institutional incentives, economy-wide security, financial institution security and general financial information showed statistical significance at 5% level, after they were regressed against the log of the amount saved for pension as the dependent variable. The latent variables were named based on items that loaded highly on each component, consistent with the propositions of the ITS which Beverly and Sherraden (1999) and Sherraden (2010) explain as items that enable a person to utilise and interact with institutions. Table 2 presents the regression coefficients of the extracted institutional constructs.

**Table 2:** Coefficients of institutional constructs together with demographic variables and model summary.

Model		Unstandardized Coefficients		Standardized Coefficients	T	Sig.	Collinearity Statistics	
		B	Std. Error	Beta			Tolerance	VIF
1	(Constant)	1.430	0.032		44.605	0.000		
	Access Provision	0.092	0.032	0.155	2.843	0.005∗∗∗	0.996	1.004
	Institutional Incentives	0.080	0.033	0.136	2.459	0.014∗∗	0.965	1.036
	Gen. Fin. Information	-0.073	0.033	-0.124	-2.244	0.026∗∗	0.960	1.041
	Fin. Inst. Security	-0.073	0.034	-0.123	-2.167	0.031∗∗	0.912	1.096
	Economy wide Security	0.070	0.033	0.119	2.107	0.036∗∗	0.926	1.080
1 & 2	(Constant)	1.082	0.170		6.377	0.000		
	Access Provision	0.048	0.027	0.082	1.771	0.078∗	0.957	1.044
	Institutional Incentives	0.044	0.028	0.074	1.580	0.115	0.939	1.065
	Gen. Fin. Information	-0.027	0.028	-0.047	-0.989	0.323	0.926	1.079
	Fin. Inst. Security	-0.048	0.028	-0.081	-1.700	0.090∗	0.900	1.111
	Economy wide Security	0.053	0.028	0.091	1.926	0.055∗	0.910	1.099
	Sex	-0.45	0.064	-0.337	-6.994	0.000∗∗∗	0.881	1.135
	Education	0.678	0.107	0.307	6.352	0.000∗∗∗	0.878	1.139
	Marital status	-0.215	0.104	-0.099	-2.069	0.039∗∗	0.894	1.118
	Dependents	-0.042	0.022	-0.087	-1.906	0.058∗	0.973	1.028
	Age	-0.001	0.003	-0.013	-0.276	0.783	0.964	1.037
Model	R	R Sq.		Adj. R Sq.	S.E	F	Sig.	
1	0.316	0.100		0.085	0.56534	6.764	0.000	
2	0.597	0.352		0.342	0.47947	34.880	0.000	
Both	0.621	0.385		0.365	0.47105	18.804	0.000	

Dependent Variable: logamtsave; ∗∗∗ p-value < 0.01, ∗∗ p-value < 0.05, ∗ p-value < 0.1.

Source: Field Survey (2019).

The estimated coefficient of access provision has a significant effect on pension saving. This means that the more access to MPS is extended to informal economy workers, the more they save towards their retirement. The items defining access provision (travel time, transaction charges, proximity to institutions, ease in opening account, eligibility criteria and awareness creation) are concerns raised by Afenyadu (2014), Asante (2016) and Kumah et al. (2017) as impeding access to MPS in Ghana. They explained that low access to MPS is due to low awareness level.

Similarly, the institutional incentive coefficient implies that the more incentives in the form of a combination of returns on investment, complementary services, acceptable interest rate, mortgage facilities and tax exemptions offered to informal workers, the more they are likely to save for retirement. This finding is consistent with those of other scholars like Han and Sherraden (2007) and Turner and Manturuk (2012), who concluded that incentives/disincentives affect participation in MPS schemes.

General financial information (financial information, financial calculation, pension and contribution education) exhibits a significant inverse relationship with pension saving. It means that the more general financial information given to informal economy workers, the more unwilling they are to save for retirement. Evidence by Bernheim and Garret (1996) and Han and Sherraden (2007) have been found to the effect that employees who are provided with specific financial information have higher participation levels in pension plans compared with those who received more general form of financial information.

The finding on the general financial information supports the argument raised by Lusardi and Mitchell (2007) that financial education cannot single-handedly improve individuals' financial literacy levels as well as their savings behaviour since there can be vast diversity of outcomes. However, the finding is inconsistent with the Institutional Theory of Savings because the theory, according to Sherraden (1991) and Beverly and Sherraden (1999), proposes that individuals’ financial or real asset accumulation is dependent on information offered by institutions.

Likewise, the estimated coefficient for financial institutions security inferred that the less confident informal workers feel about financial institutions and integrity in financial markets, the more willing they are to save. Mohd et al. (2010) and Wack (2014) found such occurrence as financial security risk and a threat to the pension saving outcomes. On the part of the economy-wide security, its estimated coefficient suggests that the more the economy is managed soundly with integrity in political systems, the more informal workers save for retirement. This is consistent with the ITS as Han and Sherraden (2007) and Beverly et al. (2008) described these security constructs as capable of inspiring saving and wealth accumulation for low-income earners.

The findings show that institutional mechanisms such as access provision, institutional incentives, general financial information, financial institution security and economy-wide security have consequential effect on pension savings of informal economy workers. As illustrated by the conceptual framework of the study, there are several institutional mechanisms proposed by the Institutional Theory of Saving. However, these significant latent variables of institutional mechanisms determine the accumulation of MPS wealth for better quality of life and income security in later life. The five significant latent variables of institutional mechanisms jointly explain ten percent of the total variation in the quantum of money saved in pension schemes at a time, and produced an adequate model (F statistic = 6.764, p-value = 0.000).

The study further observed that demographic variables command a lot of predictive power over the institutional constructs. Beside age, sex (Male), level of education (at least basic education), marital status (married) and number of dependents showed statistical significance at the 5 percent level. This finding is consistent with those of Han and Sherraden (2007), Curley et al. (2009) and Adzawla et al. (2015) who used them mainly as control variables in their respective studies. In Han and Sherraden (2007) study, age showed weak powers in explaining saving while Curley et al. (2009) and Adzawla et al. (2015) found that education is positively associated with pension saving. The implication is that people with at least basic education save more compared to those without education. In addition, Adzawla et al. (2015) found that males and married informal economy workers save less for pension compared to female and those not married respectively. The explanation offered was that married couples may spend more on children's expenses and will, therefore, have little or no money to save.

Table 2 also presents the combined estimated coefficients of the variables under consideration and their level of significance. The demographic variables explained 35.2 percent of the total variations in the amount saved. The model for the demographic variables was found to be adequate with an F – statistic of 34.880 and a corresponding p-value of 0.000.

Inspection of Table 2 gives an idea of the model summary for the institutional constructs, the demographic variables and the combination of the two. Even though the model was strengthened by the inclusion of the demographic variables, they reduced significantly the predictive powers of the institutional mechanisms. The change in R square was 3.3 percent. The combined institutional constructs and the demographics yielded an F-value of 18.804 with p-value of 0.000.

The study further confirmed and satisfied the assumptions underlying the estimation of the regression model. For instance, the Shapiro-Wilk test was used to diagnose normality of the residuals. The null hypothesis that the residuals are normally distributed was retained because the p-value (0.090) for the Shapiro-Wilk test statistic (0.992 with 321 degrees of freedom) was greater than the significance level (∝=0.05). In addition, all the VIF values for the independent variables were below 10, indicating that the assumption of no multi-collinearity is met.

Evidence from the key informants' interviews suggested that companies have put in place specific arrangements to increase informal economy workers’ enrolment onto MPS schemes. These arrangements included education and training, effective communication, flexible and attractive packages and mobile and accessible banking. A key informant from one of the trustees (2^nd^ November, 2019) explained that “We go to them to do workshops. There is a knowledge gap so we go to the lorry stations, unions and educate them to be on the pension scheme”. Another key informant (13^th^ November, 2019) emphasized that “We are option 9 when you dial ∗170# on the MTN mobile app. To enroll, we just need your name, address and the contribution you want to make”.

These quotes affirm the information, access and facilitation dimensions as established by the ITS, and thus support pension wealth build-up for retirement income security as depicted by the conceptual framework of this study. Although these institutional mechanisms when utilised well served better than the reverse, a key informant recounted that the irregularity of the informal economy posed a challenge regarding the type of education meted out:

“*The interesting thing about the informal sector is that it is a broad spectrum. A doctor who sells spare parts on the street is in the informal sector. The cocoa farmer, trader and the like exhibit different characteristics which make it a bit challenging*” (An informant, 2^nd^ November, 2019).

It can be deduced from the quotes that institutions make it a priority to make their clients feel comfortable, especially by giving financial education, easy access to accounts and making saving “automatic”, which is similar to findings reported by Beverly et al. (2008), Njuguna and Otsola (2011) and Matul et al. (2011). They mentioned these mechanisms provided by institutions as some of the dimensions in pension wealth accumulation. This assertion is portrayed by the conceptual framework that financial information and access provision lead to the build-up of pension wealth for later life consumption.

## 5 Conclusion and policy implications

Overall, the tier-three MPS scheme has paved the way for informal economy workers to save during their productive age and to consume at old age. Institutional access, incentives and economy-wide security were found to motivate informal economy workers to contribute to MPS. On the other hand, general financial information and security from financial institutions were found to demotivate enrolment and subsequent contributions to the scheme.

Having established that access provision encourages pension saving enrolment, it is reasonable for corporate trustees to partner with mobile telecommunication companies to streamline service delivery through the use of mobile apps. Such arrangement can help to address issues such as registration, service charge and payments which are major concerns in the service delivery chain. In addition, an instant service code from telecommunication companies could be generated for corporate trustees in order to help their clients in saving directly from their mobile money account. This reduces the issue of fraud and difficulty in accessing the pension savings.

Furthermore, corporate pension trustees should engage in specific financial retirement literacy campaigns, unlike general financial information to educate informal economy workers on the need to subscribe to MPS schemes and save for a better quality of life after retiring from work. Finally, the study recommends for corporate pension trustees to embark on revival of the role of institutions and creation of more successful institutional mechanisms to promote pension saving as well as asset accumulation among populations that generally do not have access to institutionalized saving mechanisms. This would address the rate of low enrolment and pension contributions of informal economy workers. This can be done by increasing security, incentives and providing specific financial information on retirement savings.

## 6 Strength and limitations

This study contributes to the discussion on extending coverage of micropension savings to informal economy workers focusing on institutional mechanisms that could make this possible. In addition, the study is unique in the sense that it made use of both quantitative and qualitative approaches to produce comprehensive and robust findings. Despite its uniqueness, the study acknowledged a sampling challenge. The results of the study were based on a cross-section of data that shows different segments of informal work groups at the same time. This could have introduced bias in the general conclusion of the entire IWC of MPS because of differences in group characteristics and size. However, this challenge was addressed using data triangulation and cross-examination with information provided by key informants.

## Declarations

### Author contribution statement

Dominic Buer Boyetey: Conceived and designed the experiments; Performed the experiments; Analyzed and interpreted the data; Wrote the paper.

Owusu Boampong: Conceived and designed the experiments.

Francis Enu-Kwesi: Contributed reagents, materials, analysis tools or data.

### Funding statement

This research did not receive any specific grant from funding agencies in the public, commercial, or not-for-profit sectors.

### Data availability statement

Data will be made available on request.

### Declaration of interests statement

The authors declare no conflict of interest.

### Additional information

No additional information is available for this paper.

## Appendix A Supplementary data

DBB_Supplementary Table DBB_Supplementary Table
