# Supplementary material for: Effect of institutional mechanisms on micropension saving among informal economy workers in the Greater Accra Region of Ghana
Source: Heliyon. 2021 Sep 16;7(9):e08004. doi: 10.1016/j.heliyon.2021.e08004 (PMC8461340; doi:10.1016/j.heliyon.2021.e08004)
Supplement: DBB_Supplementary Table [file mmc1.docx]

**APPENDIX A**

**Table 2: Factor loadings from Principal Component Analysis**

| Items | Extracted Components | | | | | | | | | | |
| --- | --- | --- | --- | --- | --- | --- | --- | --- | --- | --- | --- |
|  | 1 | 2 | 3 | 4 | 5 | 6 | 7 | 8 | 9 | 10 | 11 |
| Travelling time and cost favored your consideration to enroll in a saving scheme | 0.796 |  |  |  |  |  |  |  |  |  |  |
| Your proximity to financial institution motivate you to save | 0.792 |  |  |  |  |  |  |  |  |  |  |
| The ease in opening account motivated you to join a saving scheme | 0.724 |  |  |  |  |  |  |  |  |  |  |
| Favorable transaction charges help you to save | 0.701 |  |  |  |  |  |  |  |  |  |  |
| You easily satisfy the eligibility criteria to a saving scheme | 0.662 |  |  |  | 0.418 |  |  |  |  |  |  |
| Financial institutions create awareness of pension products to encourage savings | 0.602 |  |  |  |  |  |  |  |  | 0.567 |  |
| Having a better investment return fosters saving |  | 0.871 |  |  |  |  |  |  |  |  |  |
| Enjoying complimentary services like health insurance and access to loans motivates saving |  | 0.738 |  |  |  |  |  |  |  |  |  |
| The interest rate earned on savings is acceptable |  | 0.643 |  |  |  |  |  |  |  |  |  |
| Entitlement to a mortgage facility encourages saving |  | 0.628 |  |  |  |  |  |  |  |  |  |
| The tax exemption component on saving scheme is motivating |  | 0.556 |  |  | 0.352 |  |  |  |  |  |  |
| Pension saving providers are appealing especially, staff treatment, supports and “automatic” enrolment |  |  | 0.863 |  |  | 0.332 |  |  |  |  |  |
| Your contributions are secured |  |  | 0.863 |  |  | 0.332 |  |  |  |  |  |
| The rules regarding withdrawals are acceptable |  | 0.401 | 0.754 |  |  |  |  |  |  |  |  |
| You save more because the claim payment procedure are simple |  |  |  | 0.95 |  |  |  |  |  |  |  |
| Prompt feedbacks from financial institutions make you save more |  |  |  | 0.95 |  |  |  |  |  |  |  |
| Periodic financial statements offered motivates your savings |  |  |  | 0.741 |  |  |  |  |  |  |  |
| The financial information you received enabled you to choose an appropriate pension plan |  |  |  |  | 0.757 |  |  |  |  |  |  |
| Tutorials on simple financial calculations help you to save | 0.38 |  |  |  | 0.732 |  |  |  |  |  |  |
| Education on how to make contribution inspired your saving |  | 0.383 |  |  | 0.548 |  |  |  |  |  | 0.382 |
| You receive pension education which inspires saving | 0.411 |  |  |  | 0.534 |  |  |  |  |  |  |

**Table A1 continued.**

| Items | Extracted Components | | | | | | | | | | |
| --- | --- | --- | --- | --- | --- | --- | --- | --- | --- | --- | --- |
|  | 1 | 2 | 3 | 4 | 5 | 6 | 7 | 8 | 9 | 10 | 11 |
| Information on the benefit of pension savings encourages you to saving |  |  |  |  |  | 0.947 |  |  |  |  |  |
| Education on how to compute the return on your investment motivates you to save |  |  |  |  |  | 0.946 |  |  |  |  |  |
| You had a reward after meeting a saving target |  |  |  |  |  |  | 0.938 |  |  |  |  |
| Promotional rewards/prizes motivate saving |  |  |  |  |  |  | 0.938 |  |  |  |  |
| You are encouraged to save because of monetary incentive from institution |  |  |  |  |  |  |  | 0.945 |  |  |  |
| Gifts from institutions entice you to open account |  |  |  |  |  |  |  | 0.945 |  |  |  |
| You are confidence your institution will manage all kinds of investment risks |  |  |  |  |  |  |  |  | 0.973 |  |  |
| There is integrity in the financial market/Iinstituions |  |  |  |  |  |  |  |  | 0.973 |  |  |
| Sound management of the economy boost my confidence to save |  |  |  |  |  |  |  |  |  | 0.825 |  |
| There is integrity in the political system | 0.356 |  |  |  |  |  |  |  |  | 0.808 |  |
| Your institution is the safe place to keep money |  |  |  |  |  |  |  |  |  |  | 0.733 |
| Rules regarding borrowing/loans are acceptable | 0.469 |  |  |  |  |  |  |  |  |  | 0.628 |

Source: Field Survey (2019)

Extraction Method: Principal Component Analysis

Rotation: Varimax 0.5 loading method
